# The bacterial microbiome and metabolome in caries progression and arrest

**DOI:** 10.1080/20002297.2021.1886748

**Published:** 2021-06-16

**Authors:** Thamirys da Costa Rosa, Aline de Almeida Neves, M. Andrea Azcarate-Peril, Kimon Divaris, Di Wu, Hunyong Cho, Kevin Moss, Bruce J. Paster, Tsute Chen, Liana B. Freitas-Fernandes, Tatiana K. S. Fidalgo, Ricardo Tadeu Lopes, Ana Paula Valente, Roland R. Arnold, Apoena de Aguiar Ribeiro

**Affiliations:** a Department of Pediatric Dentistry, Fluminense Federal University, Nova Friburgo, Brazil; b Department of Pediatric Dentistry, Rio de Janeiro Federal University, Brazil; c Centre for Oral Clinical and Translational Sciences, King’s College London, London, UK; d Microbiome Core Facility, University of North Carolina School of Medicine, Chapel Hill, USA; e Department of Medicine, Division of Gastroenterology and Hepatology, School of Medicine, University of North Carolina, Chapel Hill, USA; f Division of Pediatric and Public Health, Adams School of Dentistry, University of North Carolina, Chapel Hill, USA; g Department of Epidemiology, Gillings School of Global Public Health, University of North Carolina, Chapel Hill, USA; h Division of Oral and Craniofacial Health Sciences, School of Dentistry, University of North Carolina, Chapel Hill, USA; i Department of Biostatistics, Gillings School of Global Public Health, University of North Carolina, Chapel Hill, USA; j Department of Microbiology, Forsyth Institute, Cambridge, USA; k Department of Oral Medicine, Infection and Immunity, Harvard School of Dental Medicine, Boston, USA; l National Center for Nuclear Magnetic Resonance, Federal University of Rio de Janeiro, Rio de Janeiro, Brazil; m Department of Preventive and Community Dentistry, School of Dentistry, Rio de Janeiro State University, Brazil; n Laboratory of Nuclear Instrumentation, Federal University of Rio de Janeiro, Rio De Janeiro, Brazil; o Division of Diagnostic Sciences, Adams School of Dentistry, University of North Carolina, Chapel Hill, USA

**Keywords:** Dental caries, microct, oral bacterial microbiome, metabolome, Bacteria

## Abstract

**Aim**: This *in vivo* experimental study investigated bacterial microbiome and metabolome longitudinal changes associated with enamel caries lesion progression and arrest.

**Methods**: We induced natural caries activity in three caries-free volunteers prior to four premolar extractions for orthodontic reasons. The experimental model included placement of a modified orthodontic band on smooth surfaces and a mesh on occlusal surfaces. We applied the caries-inducing protocol for 4- and 6-weeks, and subsequently promoted caries lesion arrest via a 2-week toothbrushing period. Lesions were verified clinically and quantitated via micro-CT enamel density measurements. The biofilm microbial composition was determined via 16S rRNA gene Illumina sequencing and NMR spectrometry was used for metabolomics.

**Results**: Biofilm maturation and caries lesion progression were characterized by an increase in Gram-negative anaerobes, including *Veillonella* and *Prevotella. Streptococcus* was associated caries lesion progression, while a more equal distribution of *Streptococcus, Bifidobacterium, Atopobium, Prevotella, Veillonella, and Saccharibacteria (TM7)* characterized arrest. Lactate, acetate, pyruvate, alanine, valine, and sugars were more abundant in mature biofilms compared to newly formed biofilms.

**Conclusions**: These longitudinal bacterial microbiome and metabolome results provide novel mechanistic insights into the role of the biofilm in caries progression and arrest and offer promising candidate biomarkers for validation in future studies.

## Introduction

Dental caries remains the most prevalent disease in humans worldwide and can cause pain, distress, and reduce the quality of life throughout one’s lifespan [1–4]. Recent data from the US Centers for Disease Control and Prevention (CDC) show that by age 34, more than 80% of the US population has had at least one cavitated tooth [4]. On average, the US spends more than 124 USD billion a year on dental care with more than 6 USD billion of work productivity lost each year due to dental care [5].

Caries is a diet driven, biofilm-mediated disease with distinct microbial compositions associated with its initiation, progression, and arrest that are directly dependent on individuals’ fermentable carbohydrate consumption [6]. The development of caries lesions is localized to susceptible areas for biofilm accumulation, i.e. in the pits and fissures on occlusal surfaces and proximal to the gingival margin on smooth surfaces [7,8]. Biofilm accumulation is also enhanced during tooth eruption due to reduced mechanical oral function and a reduction in cleaning efficiency, leading to a higher caries experience on molars [9–11]. Thus, it is well-established that the accumulation and maturation of a specific biofilm driven by consumption of free sugars are the key etiological factors in the development of caries lesions on occlusal and smooth surfaces, that leads to an imbalance of bacterial metabolic activity causing changes in the local environmental conditions, e.g. lower pH [7,10,12].

Several oral bacterial species have been identified and many are still ‘unculturable,’ and are known only by their genetic signatures. However, a gap remains in our understanding of the primary microbial composition and activity shifts associated with enamel caries lesion initiation, progression, and arrest [13–15]. Cross-sectional microbiome studies have shown important differences in the bacterial composition of the biofilm between different sites of the oral cavity and even between different surfaces of the same tooth [16, 17,18]. The application of high throughput technologies has also revealed the high complexity and diversity of the oral microbiome related to caries experience and activity. Our previous efforts to define the bacterial composition of the dental biofilm associated with caries activity in enamel revealed significant differences in the microbiome diversity and abundance, when comparing sound surfaces and caries lesions: among the 723 taxa identified, statistically significant differences in the relative abundances of 18 species were associated with the presence of caries lesion activity. We also showed that, among patients with high frequency of carbohydrate intake (more than two times between meals), a statistically significant increase in the relative abundances was observed among *Streptococcus sp._Oral_Taxon_487* [19].

Indeed, the integration of multiple ‘omics’ strategies with in vivo polymicrobial models may be a powerful tool to identify synergistic interactions that modulate the microbial metabolism. In this context, metabolomics is a comprehensive assessment of endogenous and exogenous low molecular weight metabolites in a biological sample which may enable understanding of stress and adaptive conditions in the oral environment. Previous studies demonstrated a shift of salivary metabolite profiles in conditions of dental caries activity and after restorative treatment, when metabolites related to microbial metabolism such as butyrate, acetate, and propionate were reduced after dental treatment [20,21]. However, there are no studies associating the bacterial microbiome of the dental biofilm, its metabolomic profile, and its influence on dental demineralization and remineralization processes.

Despite recent research on the topic of biofilm composition and dysfunction in relation to some diseases such as dental caries and periodontitis, most bacterial microbiome analyses have been cross-sectional [13,22–24]. Consequently, additional longitudinal studies are needed to associate biofilm bacterial microbiome composition and its predictive value in relation to caries activity, development and arrest. This knowledge will help to discriminate patients in need of implementation of strategies towards caries prevention, as well as to determine chairside risk assessment and effective disease management. Thus, the aim of this study was to conduct a longitudinal *in vivo* study to evaluate the bacterial microbiome and metabolome shifts associated with caries progression, and arrest, on smooth and occlusal surfaces.

## Methods

### Study population

This is a longitudinal, experimental study. Three healthy volunteers (13 years old male, 16 years old female and 20 years old male), scheduled for extractions of four premolars as part of their orthodontic treatment plan, were included. All subjects were examined using a standardized clinical protocol by a calibrated examiner (TR). All subjects presented with good oral hygiene, no signs of gingivitis and no active caries lesions, according to the visual-tactile criteria described by Nyvad et al [8]. The exclusion criteria were: current smokers, pregnancy, lactation, antibiotic therapy in the previous three months, systemic conditions that would require premedication and presence of restoration in the surface of interest. The protocol for this investigation was approved by the Institutional Review Board of Fluminense Federal University (CAAE 61649616.0.1001.5626) and informed consent was obtained from each subject and their guardians (for minor subjects).

### Caries initiation, progression, and arrest protocol

To develop the protocol, we conducted a pilot study with one patient, to test the earliest time that the lesion would be clinically detected, and that the amount of biofilm collected would be satisfactory for both microbiome and metabolome analysis. We extracted the first tooth after 3 weeks, and the other teeth in the following weeks. Clinically, after 4 weeks, the surface had lost its normal luster and appeared dull-whitish, and the biofilm collected had good readings from both microbiome and metabolome analyses.

**Figure 1.**
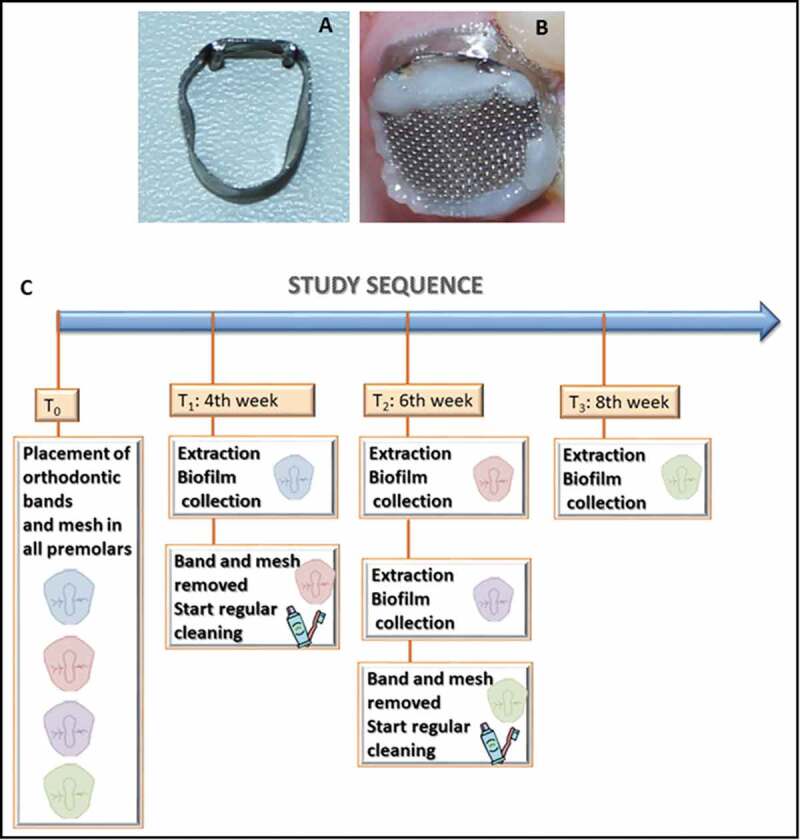
**Study model and workflow for caries lesion initiation, progression, and arrest *in vivo***. Appliances used for biofilm accumulation: (A) modified orthodontic band for smooth surfaces, and (B) mesh for occlusal surfaces. (C) Study workflow

Caries lesions initiation, progression and arrest on the premolars were induced for a maximum of eight weeks using modified orthodontic bands, i.e. with a gap on the smooth (buccal) surface, and a mesh on the occlusal surface to enable biofilm accumulation, as previously described [25–27] (Figures 1A and 1B). The teeth were extracted according to a timeline that allowed either four or six weeks of undisturbed biofilm accumulation for enamel caries lesion progression followed by a two-week mitigation period (via toothbrushing with fluoridated toothpaste) for caries lesion arrest. The subjects were instructed to continue use of their regular fluoridated toothpaste and to avoid brushing the protected surfaces only while the appliances were in place to accumulate biofilm. Volunteers were also asked not to use anti-plaque agents and not to drink black tea during the experimental period to avoid additional fluoride exposure and received no further instructions regarding diet or brushing habits. No additional cariogenic challenge was implemented (e.g. supplementation with sucrose).

The study sequence was: T0 – Installation of biofilm accumulation accessories on all teeth; T1 (fourth week) – extraction of one of the first premolars (4 W: four weeks of biofilm accumulation) and removal of the band and mesh of another premolar, so that it could be exposed to mechanical cleaning for two weeks (by conventional toothbrushing); T2 (sixth week) – extraction of the element without the appliances (4 W + 2 W: four weeks of biofilm accumulation plus two weeks of mechanical cleaning), extraction of one of the premolars (6 W: six weeks of biofilm accumulation) and removal of the devices of a third premolar; T3 (eighth week) – extraction of the remaining tooth without any biofilm accumulation accessories (6 W + 2 W: four weeks of biofilm accumulation plus two weeks of mechanical cleaning). (Figure 1C)

### Clinical evaluation and biofilm collection

At T0, the occlusal and smooth surfaces from the premolars selected for the study were diagnosed as sound, showing normal enamel translucency and texture, according to Nyvad et al [8].

All appointments for tooth extractions were carried out two hours after eating in the morning, to control the biofilm collection, according to the Manual of Procedures for Human Microbiome Project (http://hmpdacc.org/resources/tools_protocols. php). After tooth extraction, the roots were carefully cleaned with sterile gauze, the tooth was dried with a gentle air stream, the biofilm accumulation appliances were carefully removed and placed in individual Eppendorf tubes containing 100 µl of phosphate/sodium azide buffer and stored at −20°C prior to processing for metabolome analyses. Biofilm was carefully removed from occlusal and smooth surfaces of the extracted teeth, using a sterile microbrush applicator (Microbrush®) for each surface, and placed in separate Eppendorf tubes containing 100 µl of sterile transport medium (Anaerobic Dental Transport Medium; Anaerobe Systems), and frozen until processing for bacterial microbiome analysis. For sample collection from teeth submitted to mechanical cleaning (thus, with the orthodontic appliances removed at T1 and T2), patients were instructed to refrain from brushing for six hours prior to tooth extraction to allow for biofilm to form. From these teeth without appliances, biofilm was carefully removed from one half of the occlusal and smooth surface for bacterial microbiome analysis, and biofilm from the other half was used for metabolome analysis, using the same method as described above.

Clinical evaluation of the extracted teeth was conducted by a blinded and previously calibrated examiner (TR). Adopting the criteria of the Caries Activity Index proposed by 8, initial lesions were diagnosed as active white spots if the enamel surface was whitish/yellowish opaque with loss of luster, felt rough when the tip of the probe was moved gently across the surface, and had no clinically detectable loss of substance (i.e. cavitation). Occlusal surfaces should have intact fissure morphology with lesion extending along the walls of the fissure (score 1 according to the Caries Activity Index). Initial lesions were diagnosed as arrested (inactive) white spots if enamel surface was whitish, brownish, or black. Enamel was shiny and felt hard and smooth when the tip of the probe was moved gently across the surface, with no clinically detectable loss of substance (no cavitation, score 4 according to the Caries Activity Index). Then, each tooth was placed in a separate container with a humid sponge and refrigerated until micro-CT analysis.

### MicroCT analysis: image acquisition and density profile of tooth surfaces

The extracted teeth were wrapped in Parafilm™ to prevent desiccation and were subsequently scanned by a high-energy micro-CT (Skyscan 1173, Bruker, Kontich, Belgium). Acquisition parameters were defined as follows: 70kV, 114 µA, detector size 2240 × 2240 pixels, 8.19 µm pixel size, 1 mm Al filter thickness, 1s exposure time, 0.5° rotation step at 360°, average frame of 5 and random movements of 40. Scanning time was approximately 85 minutes for each specimen. After acquisition, cross-sectional images were reconstructed using a dedicated software (NRecon, Bruker) with standardized parameters, including ring artifact correction of 5, 75% beam hardening correction, no noise reduction filters and minimum (0) and maximum (0.15) contrast limits.

**Figure 2.**
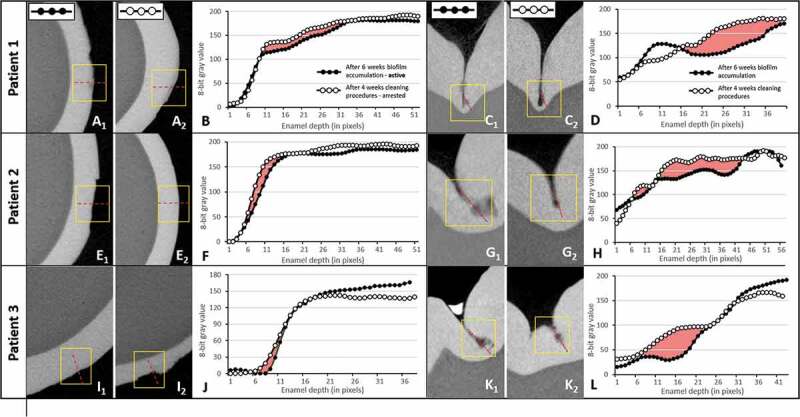
**Micro-CT analysis confirms caries lesion progression and arrest of the studied specimens in the *in vivo* model**. (A, E, I) Micro-CT cross-sectional slices of teeth after 4 weeks of biofilm accumulation (1) or after 2 weeks of tooth cleaning procedures (2) in smooth (buccal) surfaces. (C, G, K) Micro-CT cross-sectional slices of teeth after 4 weeks of biofilm accumulation (1) or after 2 weeks of tooth cleaning procedures (2) in occlusal surfaces. (B, D, F, H, J, L) Density profile plots along dotted red lines in active and arrested caries specimens. Red areas indicate ΔZ (integrated mineral loss area)

The mineral densities of the sound and caries enamel surfaces were evaluated by comparing gray value density profiles. The reconstructed image sets were imported into a 3D visualization software interface (DataViewer, Bruker) and a volume of interest (VOI) at the center of the caries lesion (approximately 2.5 mm x 2.5 mm x 2.5 mm) was selected and imported into the ImageJ software interface (FIJI implementation). Cross-sectional images were used for smooth surface lesions and sagittal cuts were used for occlusal surface lesions (Figure 2). Profiles were taken at each pixel from the enamel surface to a depth of 250 μm in the enamel layer. From the density profiles, the mineral loss parameter ΔZ was calculated and a descriptive analysis of each specimen was performed [28].

### Bacterial microbiome analysis: DNA isolation and 16S rRNA gene library preparation, sequencing, and bioinformatics analysis

Biofilms were dispersed from the micro brush by vortexing for 10 minutes. DNA isolation, preparation of sequencing libraries and sequencing were done in the UNC-Chapel Hill Microbiome Core Facility as previously described [29]. Briefly, bacterial DNA extraction was performed using QIAmp DNA extraction kit (QIAGEN). A step of pre-incubation with lysozyme for 30 min was introduced to the protocol to ensure optimal DNA yield from Gram-positive bacteria. For generation of sequencing libraries, 12.5ng of total DNA from each sample were amplified using the 2x KAPA HiFi HotStart ReadyMix (KAPA Biosystems, Wilmington, MA). Primers targeting the V3–V4 region of the 16S rRNA gene [30,31] were designed to incorporate Illumina compatible sequencing adaptors. The complete sequences of the primers were: 515 F – 5’ TCGTCGGCACGTCAGATGTGTATAAGAGACAGGTGCCAGCMGCCGCGGTAA 3’; 806 R – 5’GTCTCGTGGGCTCGGAGATGTGTATAAGAGACAGGGACTACHVGGGTWTCTAAT 3’. PCR conditions and Illumina sequencing were performed as previously described [19].

Raw sequences reads in FASTQ format were subject to DADA2 amplicon sequence variant (ASV) analysis [32] using version 1.9.1. All processed unique reads were submitted to BLASTN against 16S rRNA genes, using the BLASTN parameters: -q-5-r4-G5-E5. BLASTN results were parsed using the criteria previously published by [33]. ASVs were BLASTN-searched against a combined set of 16S rRNA reference sequences that consist of the HOMD (version 15.1 http://www.homd.org/index.php?name=seqDownload&file&type=R), HOMD 16S rRNA RefSeq Extended Version 1.1 (EXT), GreenGene Gold (GG) (http://greengenes.lbl.gov/Download/Sequence_Data/Fasta_data_files/gold_strains_gg16S_aligned.fasta.gz), and the NCBI 16S rRNA reference sequence set (ftp://ftp.ncbi.nlm.nih.gov/blast/db/16SMicrobial.tar.gz).

The NCBI BLASTN version 2.7.1+ [34] was used with the default parameters. Reads with ≥ 98% sequence identity to the matched reference and ≥ 98% alignment length (i.e. ≥ 98% of the read length that was aligned to the reference and was used to calculate the sequence percent identity) were classified based on the taxonomy of the reference sequence with highest sequence identity. If a read matched with reference sequences representing multiple species equally (i.e. equal percent identity and alignment length), it was subject to chimera checking. A multispecies assignment was considered when a read matched with reference sequences representing multiple species equally. For example, an assignment as ‘Streptococcus multispecies spp93’ indicated that a group of reads had equal top hits to references of multiple species, with identical percent identity and alignment score. These species can be looked up in the original data provided online.

All assigned reads were subject to down-stream bioinformatics analyses, including alpha and beta diversity assessments, included in the QIIME (Quantitative Insights Into Microbial Ecology) software package version 1.9.1 [35]. The phylogenetic tree required for constructing the UniFrac-based matrices used in some of the beta diversity analyses, was built dynamically from reference sequences with matched reads (no novel species identified in the de novo OTU calling stage were included in the tree due to the lack of full-length sequences). The reference sequences were aligned with the software MAFFT version 7.149b [36] prior to tree construction using the QIIME treeing script. Downstream analyses were done for a range of minimal read count per OTU/species (MC): 1, 10, and 100 separately. Original QIIME results are available on the Human Oral Microbiome Database FTP site at http://www.homd.org/ftp/publication_data/20161012/. Shannon’s index was recorded and compared at 10,000 reads/sample. Phylogenetic and non-phylogenetic beta diversity matrices were visualized using three-dimensional Principal Coordinate Analysis (PCoA) plots and calculated within QIIME using unweighted UniFrac distances between samples. Rank analyses were performed for each instance (surface – time point combination) using mean abundances of the species, averaged across the three patients. Then the ranks of mean abundances were calculated.

### Metabolomic analysis

The biofilm samples were analyzed for metabolites at the National Center for Nuclear Magnetic Resonance (CNRMN), in Brazil. Briefly, NMR analyses were performed on a 500 MHz NMR spectrometer. For polar phase analysis, in each NMR tube, 550 μl of biofilm suspension in phosphate buffer and 50 μl D_2_O (deuterated water) and 10 μl 20 mM DSS (4,4-dimethyl-4- silapentane-1-sulfonic acid) was used. The samples were analyzed by ^1^H probe using the Carl-Purcell-Meiboom-Gill (CPMG) pulse sequence with 1024 scans and ^1^H −1H TOCSY at 298 K and spectra was obtained. After spectra acquisition, edge effects were evaluated by overlaying all spectra using Topspin (Bruker Biospin). Each NMR spectrum was analyzed by integrating bucket size regions of 0.03 ppm without the water region (4.8–4.5 ppm). The bucket tables were normalized using the sum of intensities and the data were submitted to the Pareto scaling method [37] before statistical analysis. The peak intensities measured in arbitrary unity (a.u.) were analyzed in the AMIX programs (Bruker Biospin, Rheinstetten, Germany) and Metaboanalyst 2.0 (www.metaboanalyst.ca). The Partial least squares-discriminant analysis (PLS- DA) and Orthogonal PLSDA (O-PLS-DA) were performed and the determination of the relative abundances of the metabolites that contributed to the separation between the groups related to surface clinical diagnosis was analyzed by the Variable Importance in Projections (VIPs) scores [38]. Metaboanalyst 3.0 software was also used to obtain the predictive performance of the models; each model was evaluated for Q2, R2 and accuracy (ACC), for the purpose of cross-validation [39]. The assignment was performed based on the Human Metabolome database (http://www.hmdb.ca/) as in previous studies [20,21,40]. ^1^H – ^1^H total correlation (TOCSY) spectra were acquired to confirm metabolite assignments using 256 X 2,048 points, spectral width of 12,019 Hz in each dimension and a mixing time of 70 ms.

## Results

### Evaluation of enamel caries progression and arrest

All dental surfaces evaluated before the experimental procedures received a score of 0 (sound) [8], except for the occlusal surfaces of teeth #5 and #12 of patient 2, which had a restoration (score 7). Therefore, these two premolar surfaces were excluded from the study. After removal of orthodontic bands and meshes, all tooth surfaces were examined by a blinded and previously calibrated examiner (TR). All surfaces subjected to biofilm accumulation developed an active initial white spot lesion and were classified with score 1, while all surfaces submitted to toothbrushing with fluoridated toothpaste for two weeks were classified as score 4 (arrested white spot lesion) according to the Caries Activity Index [8]. Supplemental Figure S1 shows the clinical pictures of all premolars, in all phases of the study.

Although the teeth submitted to four weeks of biofilm accumulation showed clinically visible white spot lesions, these lesions were not visually identified using micro-CT and therefore, detailed density evaluations were not undertaken (Supplemental Figure S2).

After six weeks of biofilm accumulation, patient 1 exhibited an active white spot lesion on the buccal surface with an erosion pattern with evidence of some surface loss (yellow squared inset, Figure 2A_1_). After two weeks of introducing tooth brushing with fluoridated toothpaste (following the removal of the orthodontic bands and mesh), some signs of subsurface smooth surface caries lesion were still evident (Figure 2A_2_). For patients 2 and 3, enamel changes other than surface erosion were not easily visualized (Figures 2E and 2I) but the density profile graphs showed, for all patients, a slight difference in mineral density (density loss) between the caries lesion and arrested caries lesion (red areas in Figure 2B, 2F and 2J). In occlusal surfaces, initial enamel caries lesions (in the pattern of lunar-shaped lesions) were found in the middle, but more frequently at the bottom of the fissure walls (Figures 2C_1_, Figure 2G_1_ and Figure 2K_1_). Arrested occlusal lesions were less progressive than active ones, as seen by the sagittal sections (Figure 2C, 2G and 2K) and confirmed by their higher density profiles (Figure 2D, 2H and 2L). The integrated density profile areas (red areas) were larger compared to those of buccal lesions, indicating higher mineral loss in the occlusal surface lesions. In all lesions and patients, higher density profiles after the toothbrushing period supported the arrest of these caries lesions.

### The bacterial microbiome composition varies according to surface, time, lesion progression and arrest

**Figure 3.**
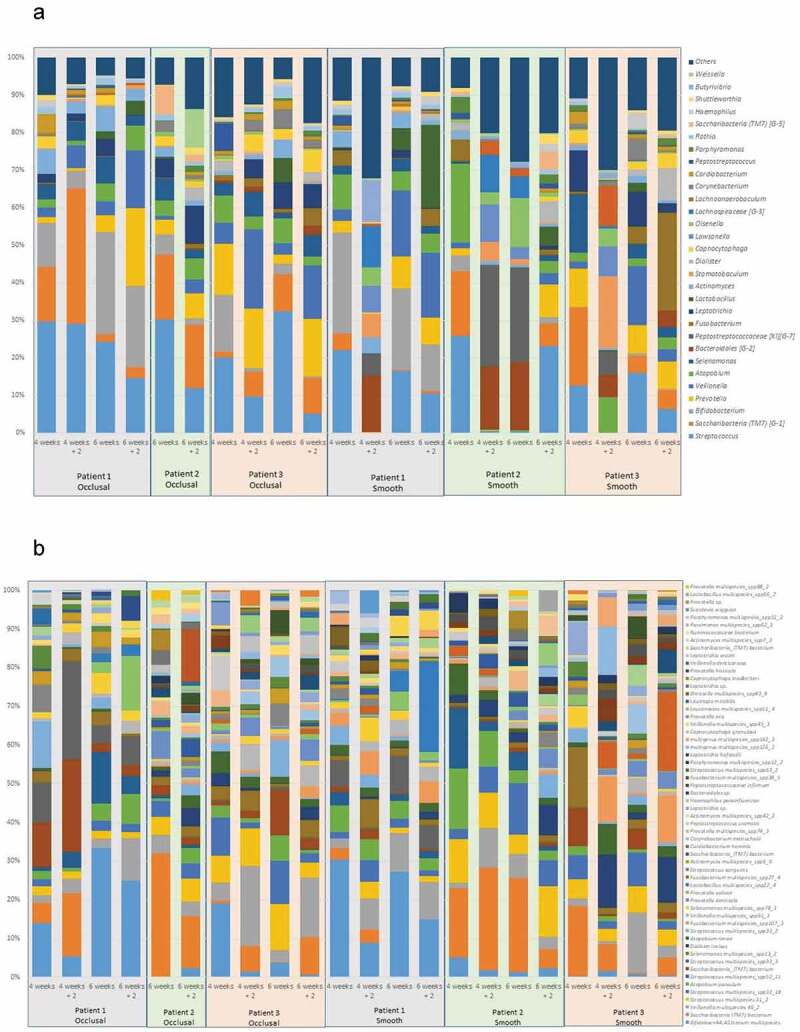
Most abundant bacterial genera (A) and species (B): profiles according to patient, timeline, and dental surfaces

A total of 3,223,479 sequences were generated from the 24 biofilm samples, and 1,436,144 reads matched 731 unique species. Amplicon reads with relative abundances higher than 0.01% were assigned to 10 bacterial phyla, 27 classes, 38 orders, 57 families, 118 genera and 340 species. Figure 3 shows the most abundant bacterial genera and species profile distribution, according to patients, time points and dental surfaces.

**Figure 4.**
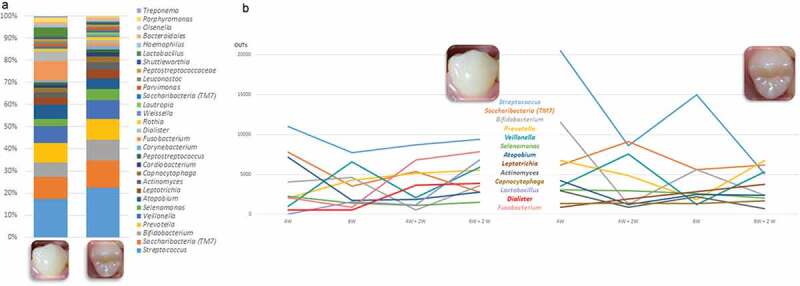
**Genus differences in the bacterial microbiome composition in relation to dental surfaces and timeline**. (A) Total distribution of bacterial taxa at genus level on smooth and occlusal surfaces. For illustrative purposes, only genera with abundances ≥ 1% on smooth or occlusal surfaces are represented. The total representation per sample is 92%. (B) Different time points show variations in relation to bacterial microbiome genus abundances (expressed by median OTUs). For illustrative purposes, only the 10 more abundant genera are represented

Comparisons of the bacterial diversity and composition among tooth surfaces showed that differences were observed between smooth and occlusal surfaces. *Streptococcus* spp. were found at a mean abundance of 16% (ranging from 5% to 23%) and 21% (ranging from 6% to 33%) on the smooth and occlusal surfaces, respectively. *Fusobacterium* was more abundant on the smooth surfaces (8%) than occlusal surfaces (1%). A summary of these compositional differences is presented in Figure 4A. The bacterial microbiome composition also varied according to time. *Streptococcus* and *Atopobium* were more abundant in mature biofilms, while *TM7* and *Prevotella* were more abundant in biofilms exposed to mechanical cleaning, in both occlusal and smooth surfaces (Figure 4B). *Campylobacter* was highly abundant in mature biofilms on occlusal surfaces, and *Peptostreptococcus* on smooth surfaces.

**Figure 5.**
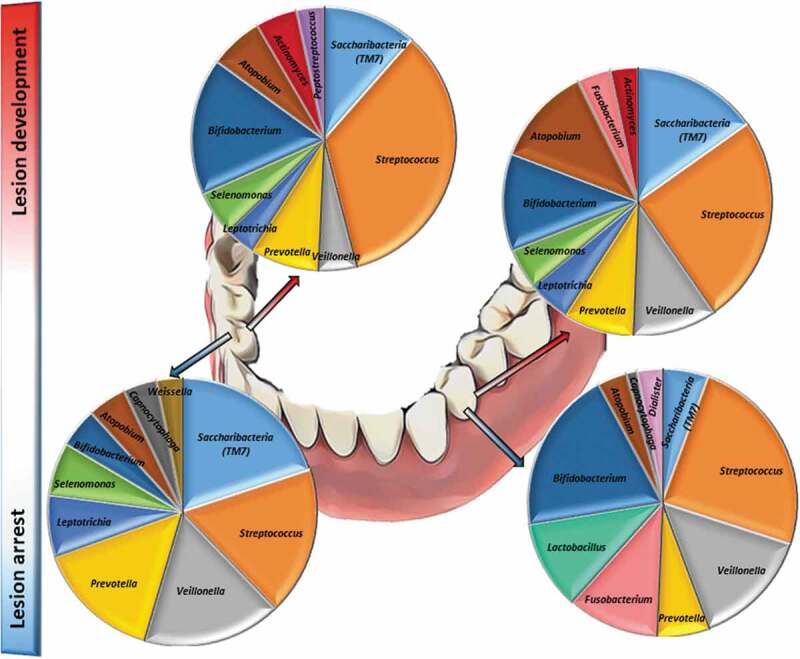
**Bacterial composition in biofilms from active and arrested caries lesions at the genus level derived from 16S rRNA gene Illumina sequencing. Median values**. Red arrows indicate lesion development (active white spot lesion, upper graphs) and blue arrows indicate lesion arrest (inactive white spot lesion, lower graphs)

This experimental model showed differences in genera abundances from occlusal and smooth surfaces during enamel caries lesion progression (considered by four weeks and six weeks of biofilm accumulation) and arrest (considered following two weeks of mechanical cleaning). Among the top 10 most abundant genera, *Streptococcus, Bifidobacterium, Atopobium, Prevotella, Veillonella*, and *Saccharibacteria (TM7)* were found in high abundance during lesion progression and arrest, on occlusal and smooth surfaces, while *Fusobacterium* was found only on smooth surfaces during lesion progression and arrest. Biofilm samples also showed that *Streptococcus* was the dominant genus among caries lesions progression (i.e. in biofilm harvested from active white spot lesions), while in biofilms from arrested caries lesions (i.e. in biofilm harvested from inactive white spot lesions) the genera composition appeared to be more equally distributed, with similar abundances as shown in Figure 5.

Table 1 shows in a color coded heatmap format that grades the mean percentages of the 10 top ranked species across all time points. Red indicates the highest mean abundance, green indicates the lowest abundance among the top ranked species. The biofilm composition after four weeks of accumulation exhibited the same 10 top ranked species in abundances ranging from 2.3% to 10.5% in both smooth and occlusal surfaces, and was composed primarily of Gram-positive facultative anaerobes, with the exception of *Selenomonas* multispecies group (*Selenomonas sputigena and/or Selenomonas sp. HMT 442)*. After six weeks, biofilm maturation and lesion progression were characterized by an increase of Gram-negative anaerobes, including *Veillonella spp*. and *Prevotella spp*. and a higher diversity in genera among the top ten ranked species. At six and eight weeks, even with the introduction of mechanical disturbance of the biofilm, anaerobic, Gram-negative species from genera such as *Fusobacterium, Veillonella* and *Prevotella* were among the top 10 most abundant species identified. *Streptococcus gordonii, Bifidobacterium* multispecies (*B. dentium, B. moukalabense), Streptococcus* multispecies (combination of two or more of the following species–*S. gordonii, S. mitis, S. oralis subsp._dentisani, S. oralis, S. oralis subsp. tigurinus, S. infantis, S. pneumoniae, S. cristatus, S. oralis subsp. oralis spp153*_*2*), and *Saccharibacteria bacterium* HMT 346 were among the top ranked abundant species at all time-points, on both smooth and occlusal surfaces. *S. mutans* accounted for 0.3% to 1% of the total community during lesion progression. Relative abundances of *Kingella oralis, Rothia dentocariosa, Gemella sp*., and *Alloprevotella sp HMT 308* were overrepresented in mature biofilms (associated with lesion progression) in comparison with newly formed biofilms (associated with lesion arrest).

**Table 1.** Top 10 ranked most abundant bacterial species in biofilms from lesion development (active white spot lesion) and arrest (inactive white spot lesion)

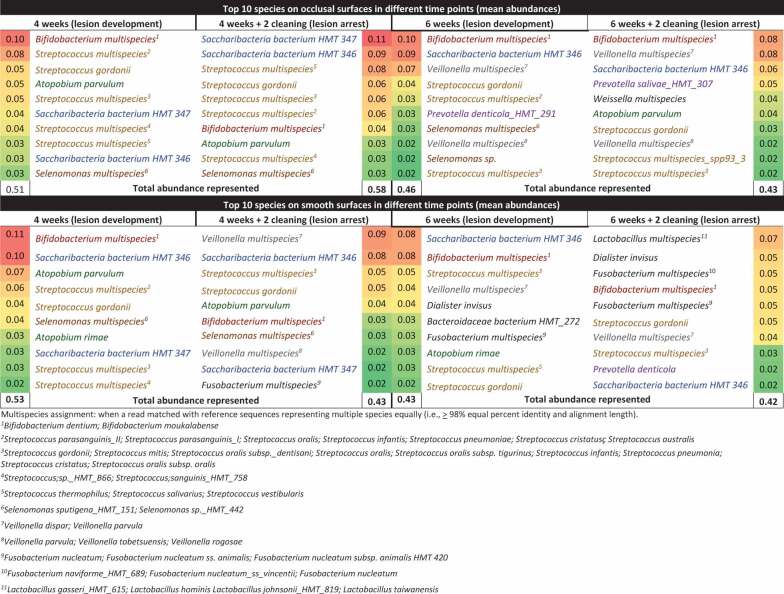

Comparison of samples between experimental timelines showed that biofilms harvested at eight weeks (6 W + 2 W: six weeks plus two weeks of mechanical intervention) had higher Shannon diversity indices from both smooth and occlusal surfaces, while lower diversity index levels were observed from occlusal biofilms from newly formed biofilms after 4 weeks of accumulation following mechanical cleaning (4 W + 2 W); and at four weeks from smooth surfaces (4 W; Supplemental Figures S3A and S3B). Principal Coordinate Analysis (PCoA) of unweighted UniFrac showed that samples did not form well defined clusters according to the time points and outcomes evaluated (Supplemental Figures S3C and S3D).

### The bacterial metabolic products are variable according to maturation stages of the biofilm

The ^1^H NMR spectra of biofilm of occlusal and smooth surfaces and after 4- and 6 weeks biofilm formation and 2 weeks arrest are shown in Supplemental Figure S4, depicting regions for metabolites such as lactate, acetate, alanine, butyrate, and sugar.

**Figure 6.**
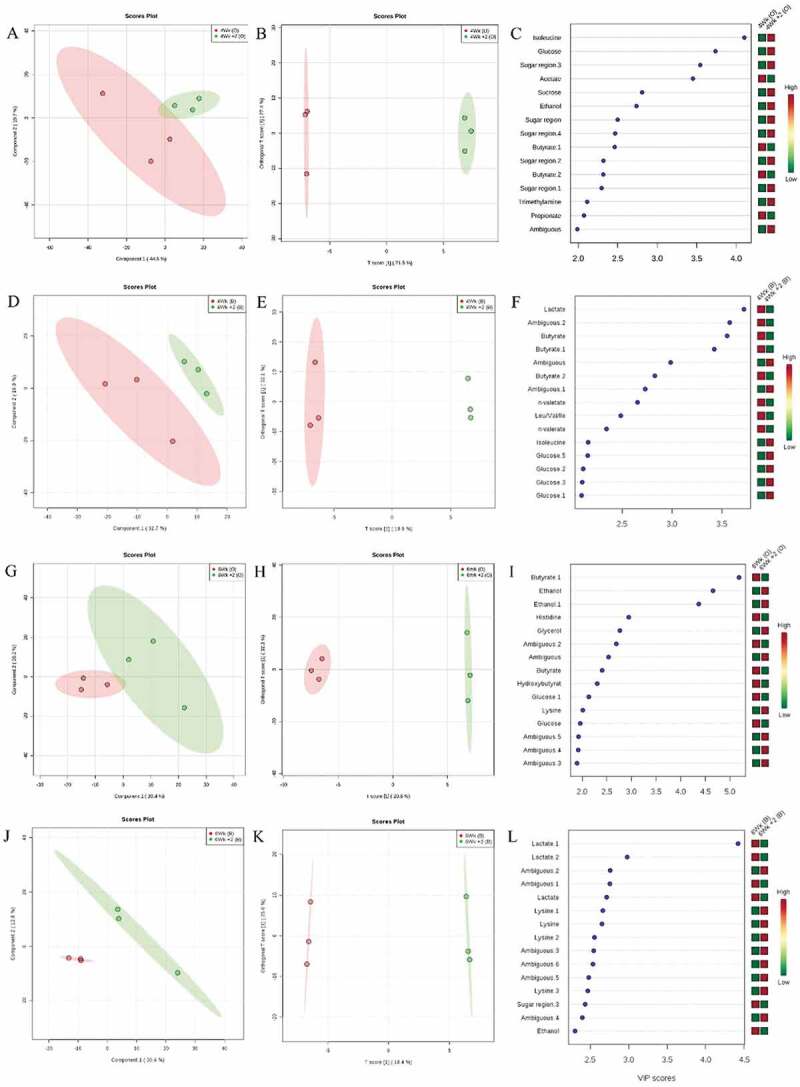
**Differences in bacterial metabolomic profiles between groups**. (A) PLSDA; (B) O-PLS-DA; (C) VIP score of 4-weeks mature biofilm and 2-weeks newly formed biofilm in occlusal surface. (D) PLSDA; (E) O-PLS-DA; (F) VIP score of 4-weeks mature biofilm and 2-weeks newly formed biofilm in buccal surface. (G) PLSDA; (H) O-PLS-DA; (I) VIP score of 6-weeks mature biofilm and 2-weeks newly formed biofilm in occlusal surface. (J) PLSDA; (K) O-PLS-DA; (L) VIP score of 6-weeks mature biofilm and 2-weeks newly formed

The metabolic profile of the bacterial biofilm was distinct when comparing the different maturation times (four versus six weeks) and of the influence of mechanical intervention. Supplemental Table S1 shows that performance of models exhibited low accuracy (from 0.16 and 0.33) and high R^2^ (from 0.96 to 0.99) when compared 4 and 6 weeks with newly formed biofilms. The multivariate statistical analysis through PLS-DA and O-PLS-DA strategies distinguished 4 weeks mature biofilms from 4 W + 2 W (newly formed biofilms following mechanical cleaning in occlusal surface; Figures 6A and 6B, respectively) and smooth surface (Figures 6D and 6F, respectively). Similarly, the 6-weeks mature biofilm exhibited differences in the metabolite profile when compared to 6 W + 2 W newly formed biofilms following mechanical cleaning in both PLS-DA and O-PLS-DA in occlusal surface (Figures 6G and 6H, respectively) and smooth surface (Figures 6I and 6J, respectively).

Figures 6C and 6F present the variable importance in projection (VIP) of 4-weeks biofilm in occlusal and buccal surfaces, respectively. Acetate, butyrate, propionate and n-valerate increased, while sugar metabolites and ethanol decreased in mature biofilm. Figures 6I and 6L show the 6-weeks mature biofilm in occlusal and buccal surfaces demonstrating higher amounts of lactate, butyrate, ethanol, and sugar region sugar metabolites in mature biofilm and reduced levels of lysine and ethanol in the newly formed biofilm.

## Discussion

The present study employed a longitudinal *in vivo* caries formation model and found that caries lesion progression was associated with biofilm maturation characterized by an increase of Gram-negative anaerobes, including *Veillonella* and *Prevotella*, and bacterial species such as *Kingella oralis, Rothia dentocariosa, Gemella sp*. and *Alloprevotella sp* HMT 308, marked by an increase in concentrations of lactate, acetate, pyruvate, alanine, valine, and sugars. The experimental design implemented here benefits in terms of offering a more accurate analysis of the caries lesion histopathology as compared to *in vitro* or *in situ* models. Our study followed the continuous process of caries lesion progression and arrest using high- throughput sequencing technology. These preliminary longitudinal bacterial microbiome and metabolome results provided novel mechanistic insights into the role of the biofilm in caries progression and arrest and offer promising candidate biomarkers for validation in future studies, that can be used as ‘risk profiles’ to predict caries development.

*In vivo* models have been previously tested for caries development [26,27] but, to our knowledge, this is the first study to use this model to investigate the biological shift during both process of enamel caries lesion progression and arrest (by including brushing), combining bacterial microbiome, metabolome, and enamel structure analyses. The design of the study was also planned to mimic the most realistic scenario. During lesion progression, the use of the biofilm accumulated in contact and adhered to the tooth surface was chosen for the microbiome analysis due to the known structure of the biofilm; while the metabolome analysis were conducted with the remaining material at the orthodontic appliances because the metabolites are diffuse at the biofilm and thus, would be retained in the biological material adhered to the appliance.

Previous studies using the same model showed that caries lesions forming under this method are histologically comparable with naturally developing lesions. Specifically, Hals and Simonsen [41] reported that the outer lesion type that developed had the same histological features, including a surface zone in polarized light, as previously described in natural “white spot” lesions by Silverstone, in 1973 [42]. Occlusal surfaces are naturally covered with plaque during the eruption period, as showed previously by Carvalho et al. [9], while teeth in occlusion shows plaque accumulation at the pit and fissure area. In our study, despite the fact that the occlusal surface Was covered by the net, its surroundings were covered by composite, to keep the net in place, thus leaving the pit and fissure area free for biofilm accumulation and, consequently, for lesion development, as shown by our micro-CT images. On smooth surfaces, the micro-CT patterns at six weeks of biofilm accumulation exhibited an active white spot lesion with an erosion pattern and evidence of some surface loss. This pattern was previously evidenced by Holmen *et al*. [43,44], who also showed typical features of active enamel lesions with surface erosion and enlarged inter crystalline pathways after biofilm removal.

It is known that the bacterial microbiome from dental biofilms can harbor more than 720 distinct species [19], since the use of more modern techniques such as sequencing enables the investigation of differences in abundance and diversity patterns across age, sample quality and origin, and health status [45]. Although many studies compared the bacterial microbiome associated with patients with and without dental caries, most of them used pooled samples (for example, saliva or pooled biofilm from multiple tooth surfaces) [16,46–48], while in the present study, comparisons of the bacterial diversity and composition among caries active and arrested sites without pooling samples from different tooth surfaces showed differences between smooth and occlusal surfaces, indicating potentially different micro niches that allow for different microbial communities, and thus, pooled samples may not reflect the real distribution of the bacterial microbiome composition on each surface. This individualized sample collection approach is desirable to avoid discrepancies in the diversity of bacterial microbiome profiles even within surfaces of the same teeth and individual (as shown in Figure 3), and as previously reported by Simón-Soro et al. [17],and Carda-Diéguez et al. [18]. This is consistent with the localized nature of the microhabitats responsible for disease development, whereas pooled sampling might obscure or ‘dilute’ the microbial composition at the involved sites.

We verified that *Streptococcus* accounted for 35% and 25% of the total community from active white spot lesions from occlusal and smooth surfaces, respectively (as shown in Figure 4). However, *S. mutans* was in low abundance and accounted for 0.3% to 1% of the total community during lesion progression, as also shown previously by Simón-Soro et al [14], with a representation of 0.02 to 0.73% of the total bacterial community. In contrast with our previous findings from active lesions on occlusal surfaces, *S. mutans* accounted for an average 7.2% of species abundance [19]. This difference may be because the present study evaluated the bacterial profile associated with the longitudinal bacterial microbiome shift during early stages of the lesion development, in contrast to our previous cross-sectional study that evaluated the bacterial microbiome community in well and naturally developed active white spot lesion on occlusal surfaces.

Our findings are aligned with recent studies that showed that, in addition to *S. mutans* and *S. sobrinus*, the list of caries-associated species include species of *Actinomyces (A. gerencseriae, A. naeslundii and A. israelii), Abiotrophia, Atopobium, Bifidobacterium, Lactobacillus, Olsenella, Pseudoramibacter, Scardovia, Selenomonas* and *Veillonella* [14,16,19,27,48]. Simón-Soro et al [14], using RNA-seq methods found that streptococci accounted for 40% of the total active community in active white spot lesions, being *Streptococcus, Rothia, Leptotrichia* and *Veillonella* the dominant genera observed among active white spot lesions. We found that *Saccharibacteria* (formerly *TM7*) and *Bifidobacterium* were highly abundant in the biofilm community associated with lesion activity in enamel, together with *Streptococcus* and *Veillonella*. There are no previous studies examining bacterial microbiome community profiles’ association with lesion arrest. But since it is known that to inactivate a lesion, it is necessary to constantly remove or disturb the biofilm by mechanical cleaning, we compared our findings with previous results from newly formed biofilms, which showed that *Streptococcus, Prevotella* and *Veillonella* are among the most abundant taxa at the genus level [49–51]. Our findings also corroborate the study by Simón-Soro et al. [17], where *Fusobacterium, Prevotella, Streptococcus*, and *Capnocytophaga* were among the most abundant taxa at genus level on sound smooth surfaces.

At the species level, our findings identified that *Kingella oralis, Rothia dentocariosa, Gemella sp*. and *Alloprevotella sp*. HMT 308 may be associated with a ‘danger profile’ to increased risk for caries development as also shown in other cross-sectional studies [52–54], while *Treponema denticola, Dialister pneumosintes, Campylobacter gracilis, Alloprevotella tannerae, Shuttleworthia satelles, Oribacterium sp* and *Gemella sp* were associated with the shift towards lesion arrest and, thus, could be seen as part of a ‘health conducive profile’. Interestingly, *Fusobacterium nucleatum* was associated with caries progression on occlusal surfaces, but also associated with lesion arrest on smooth surfaces. *Fusobacterium nucleatum* has been previously associated with the healthy microbiota from sound surfaces [27,55].

We also showed that newly formed biofilms (those constantly modified by surface cleaning) on smooth surfaces are characterized by higher alpha diversity, while mature biofilms are characterized by lower alpha diversity. This may be explained as special abilities are required from the microorganism to survive and to overcome a hostile environment, such as the ability to form biofilm by the synthesis of adhesive glucans from sucrose, like *S. mutans* [56]. Species other than *S. mutans*, such as *S. sobrinus, Rothia dentocariosa, Actinomyces species* and *S. salivarius* are also related to the early stages of dental caries due to the genetic virulence repertoire that allows these species to set up the environment for more acid-tolerant and acidogenic species, including *Scardovia wiggsiae* and *Actinomyces* sp. HOT 448 [54].

Since we were interested in investigating the microbial composition and metabolite shifts in the most natural and realistic manner possible, no dietary counseling and no specific oral health instructions were given during the study. However, since the present set-up allowed the subjects to continue their regular toothbrushing habits, including the use of fluoridated dentifrice, it may explain the low severity of the lesions formed, especially on the smooth surfaces even after eight weeks of cariogenic challenge (Figure 2). Besides the use of fluoridated dentifrice, the fluoride level of water supply can also influence the development of caries lesions. Although micro-CT studies allow good control of experiments, by allowing ‘before’ and ‘after’ evaluations and registering of images, as this was an *in vivo* study, specimens were not the same in each treatment exposure, and for this reason, variations in the location of the analyzed areas varied slightly between caries and arrested caries specimens. Caries lesions were deeper on the occlusal surfaces, which probably reflected on the microbiome and metabolic acid profile of this surface. Although the bacterial microbiome composition of the surfaces exhibited almost the same top 10 species, the occlusal surfaces had higher abundances of acidogenic and aciduric species such as *Streptococcus* sp.

Metabolomics can identify perturbations in biological systems that result to disease conditions and can help identify specific biochemical fingerprints of the disease cycle [57]. In the current study, the biofilm from 4- and 6-weeks resulted in increased levels of organic acids such as lactate, butyrate, and propionate in comparison to the newly formed biofilm. The mature biofilm accumulates organic acid in both occlusal and buccal surfaces, leading to the mineral loss seen at the density level. These results are in agreement with previous findings, which showed an increase of organic acids in dental caries lesions in the dentin [20,21,58]. The present work demonstrated slight difference in organic acid content between occlusal and buccal surface. These compounds are related to oral microorganism metabolism that produce organic acids by sugar fermentation, which lead to decreases in dental plaque pH that synergistically with the increasing of the porosity of the dental plaque matrix result in demineralization [59,60]. The acidic microenvironment favors acidophilic microorganisms’ growth and reinforce the demineralization cycle. During the arrest period, as expected, the amount of organic acids in the samples was reduced. The increased levels of sugar in the newly formed biofilm in comparison to four weeks biofilm can be explained by the reduced use of these carbohydrates for the immature biofilm. Conversely, the six weeks biofilm retained increased levels of carbohydrates in comparison to the newly formed biofilm. The process of biofilm maturation produces different metabolomic profiles, reflective of biofilm characteristics.

Finally, although the small number of participants studied is one of the limitations of this study, reporting of these preliminary data conveys insightful information regarding the efficacy of this model to detect both microbiome and metabolome shifts during lesion progression and arrest, and are informative for guiding future investigations. Also, our data demonstrated a model that can be used for *in vivo* studies to promote the understanding of the relationship between the function and the structural composition of these biofilms is important to elucidate the caries progression and arrest process. The identification of a microbiologic and metabolomic biofilm fingerprint can ultimately translate into a signature associated with at-risk sites with implications in caries risk assessment, caries diagnosis and caries management.

In conclusion, our findings showed that the bacterial microbiome and metabolome associated with active white spot lesion progression was discernibly different from that of the bacterial microbiome associated with white spot lesion arrest. These longitudinal results provide novel mechanistic insights into the role of the biofilm in caries progression and arrest and offer promising candidate biomarkers for validation in future comprehensive longitudinal studies, for screening and assessing the risk of caries development, which can be used in precision medicine, helping the clinicians and patients prevent the disease from establishing with timely management of the dental biofilm.

## Supplementary Material

Supplemental Material
