# Supplementary material for: The bacterial microbiome and metabolome in caries progression and arrest
Source: J Oral Microbiol. 2021 Jun 16;13(1):1886748. doi: 10.1080/20002297.2021.1886748 (PMC8211139; doi:10.1080/20002297.2021.1886748)
Supplement: Supplemental Material [file ZJOM_A_1886748_SM7898.zip › Supplementary files/Supplemental table 1.docx]

| Models | Accuracy | R^2^ |
| --- | --- | --- |
| 4-weeks and newly formed biofilm - occlusal | 0.33 | 0.97 |
| 4-weeks and newly formed biofilm - buccal | 0.16 | 0.98 |
| 6-weeks and newly formed biofilm - occlusal | 0.33 | 0.96 |
| 6-weeks and newly formed biofilm - buccal | 0.16 | 0.99 |
