# Supplementary material for: The bacterial microbiome and metabolome in caries progression and arrest
Source: J Oral Microbiol. 2021 Jun 16;13(1):1886748. doi: 10.1080/20002297.2021.1886748 (PMC8211139; doi:10.1080/20002297.2021.1886748)
Supplement: Supplemental Material [file ZJOM_A_1886748_SM7898.zip › Supplementary files/Supplementary Legend.docx]

**Supplemental Figures**

**SF1:** Clinical pictures of all premolars included, in all phases of the study.

**SF2:** Teeth submitted to 4 weeks of biofilm accumulation and corresponding micro-CT images. A) Patient 1; B) Patient 2; C) Patient 3

**SF3:** Alpha diversity (Shannon Index) of the sequence reads from occlusal (A) and smooth (B) surfaces in different time points (during caries lesion progression - 4W and 6W; and arrest – 4W + 2W and 6W + 2 W). (C and D) Principal Coordinate Analysis (PCoA) of unweighted UniFrac during caries lesion progression - 4W and 6W; and arrest – 4W + 2W and 6W + 2 W.

**SF4:** Representative ^1^H spectra of polar phase of biofilm from different tooth surfaces. A) Polar phase of 4-weeks biofilm from occlusal surface; B) Polar phase of 4-weeks biofilm and arrest after 2-weeks from occlusal surface; C) Polar phase of 6-weeks biofilm from occlusal surface; D) Polar phase of 6-weeks biofilm and arrest after 2 weeks from occlusal surface; E) Polar phase of 4-weeks biofilm from vestibular surface.

**Supplemental Table:**

**ST1:** Performance of the models
