# Supplementary figures and images for: The bacterial microbiome and metabolome in caries progression and arrest
Source: J Oral Microbiol. 2021 Jun 16;13(1):1886748. doi: 10.1080/20002297.2021.1886748 (PMC8211139; doi:10.1080/20002297.2021.1886748)

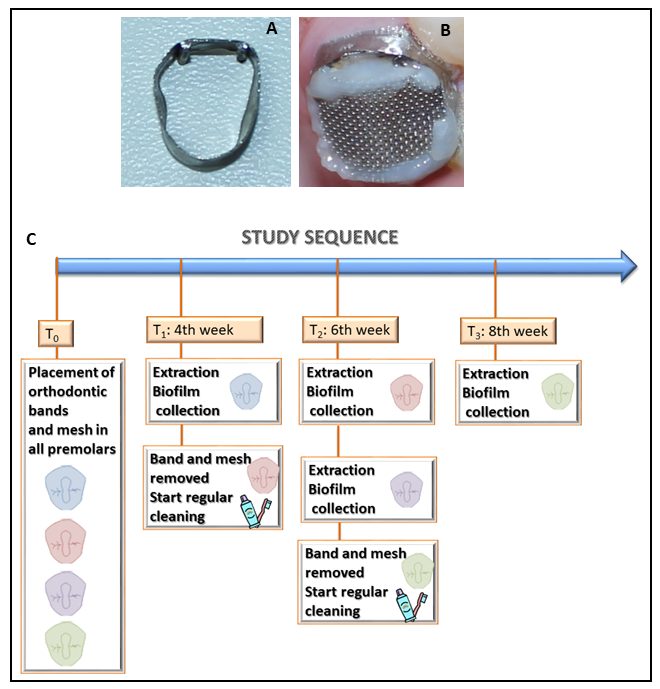

Supplement: Supplemental Material [file ZJOM_A_1886748_SM7898.zip › Supplementary files/Supplemental Figure 1.tif]

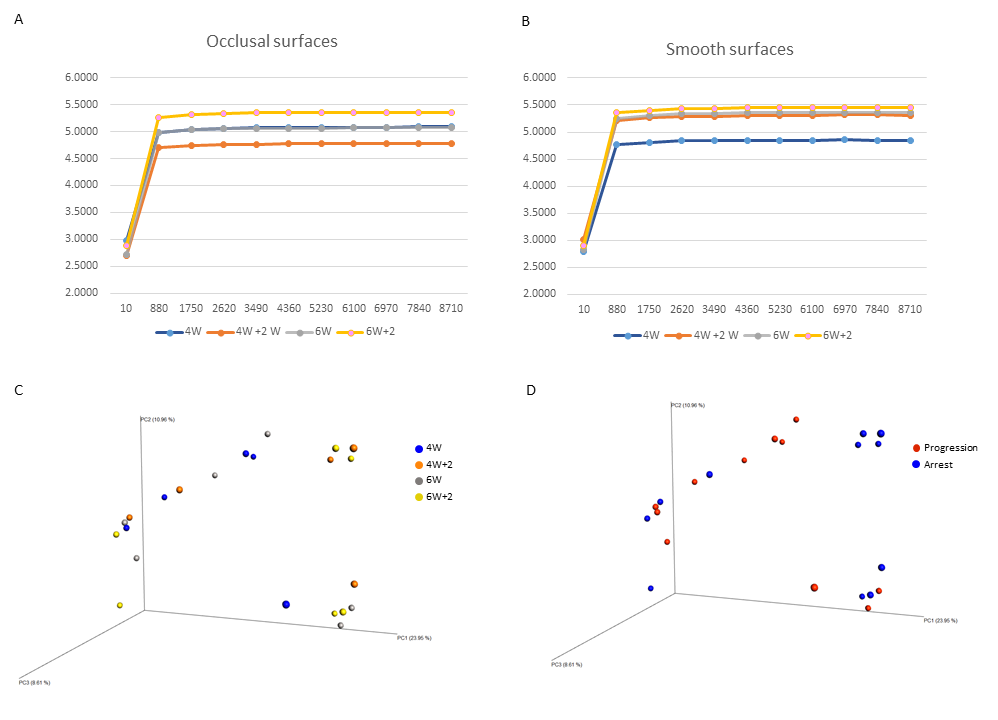

Supplement: Supplemental Material [file ZJOM_A_1886748_SM7898.zip › Supplementary files/Supplemental Figure 3.tif]

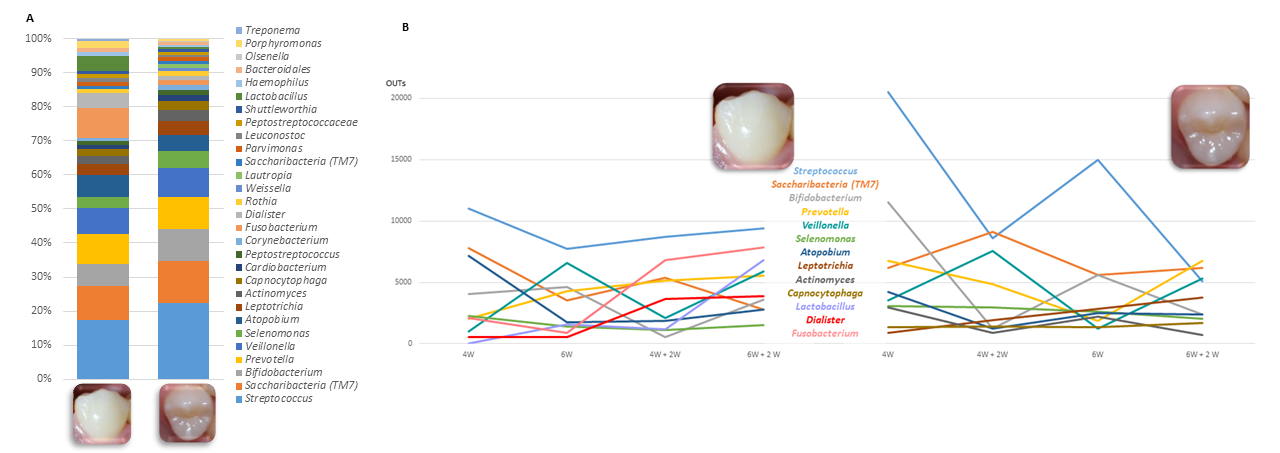

Supplement: Supplemental Material [file ZJOM_A_1886748_SM7898.zip › Supplementary files/Supplemental Figure 4.tif]

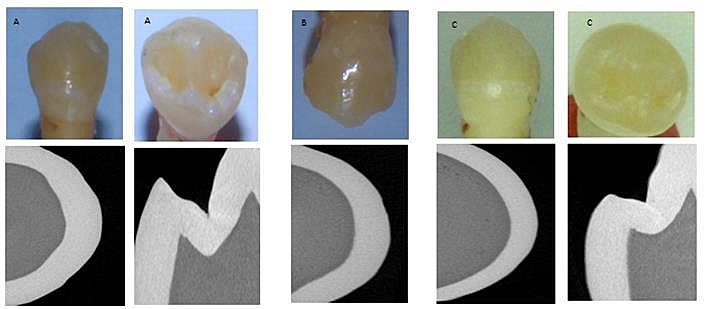

Supplement: Supplemental Material [file ZJOM_A_1886748_SM7898.zip › Supplementary files/Supplemental_figure_2.tiff]
